# Deglycosylated azithromycin alleviates cisplatin-evoked constipation in mice by altering host metabolome and gut microbiota composition

**DOI:** 10.3389/fmicb.2025.1437662

**Published:** 2025-05-21

**Authors:** Xiaoting Gu, Shuwen Zhu, Weixue Tian, Xiaohe Li, Yutian Cai, Chaoyue Zheng, Xiang Xu, Conglu Zhao, Hongting Liu, Yao Sun, Zhilin Luo, Honggang Zhou, Xiaoyu Ai, Cheng Yang

**Affiliations:** ^1^State Key Laboratory of Medicinal Chemical Biology, College of Pharmacy and Tianjin Key Laboratory of Molecular Drug Research, Nankai University, Tianjin, China; ^2^Tianjin Key Laboratory of Molecular Drug Research, Tianjin International Joint Academy of Biomedicine, Tianjin, China; ^3^The National Institutes of Pharmaceutical R&D Co., Ltd., Beijing, China

**Keywords:** Deg-AZM, pharmacodynamic, 16S rRNA, untargeted metabolomics, gut microbiota, intestinal motility

## Abstract

**Introduction:**

Chemotherapy induced constipation (CIC) is a gastrointestinal side effect that occurs in patients receiving chemotherapy, which can further deteriorate the living quality of cancer patients. Deglycosylated azithromycin (Deg-AZM), a newly developed Class I drug with good therapeutic effects on chronic constipation, has been approved for clinical trials in 2024. However, it is unclear whether Deg-AZM has any impact on gut microbiota of CIC mice. The purpose of this study was to explore the role of Deg-AZM in treating CIC by modulating the gut microbiota.

**Methods:**

The therapeutic effects of Deg-AZM on intestinal motility were assessed in a cisplatin-induced CIC mouse model. The gut microbiota composition was analyzed using 16S rRNA sequencing, and metabolic changes were evaluated through untargeted metabolomics of fecal samples.

**Results:**

Deg-AZM significantly enhanced intestinal motility in the mice with cisplatin-evoked constipation. Gut microbiota analysis revealed that Deg-AZM altered the community composition by decreasing *Deferribacterota* and *Pseudomonadota* and increasing *Bacteroidota*, *Lactobacillus* and *Muribaculaceae*. The feces metabolomics revealed that alanine, aspartate and glutamate metabolism, citrate cycle (TCA cycle), purine metabolism, primary bile acid biosynthesis and taurine and hypotaurine metabolism in CIC model were modulated by Deg-AZM.

**Conclusion:**

Deg-AZM could alleviate cisplatin-evoked constipation in mice by reshaping the structure of gut microbial community, which may provide a potential basis for the use and clinical development of Deg-AZM for CIC treatment.

## 1 Introduction

Chemotherapy induced constipation (CIC) is a chronic constipation phenomenon in cancer patients after receiving chemotherapeutic drugs or related auxiliary treatments (Escalante et al., 2017). Reports indicated that the incidence rate of chemotherapy-induced constipation in cancer patients ranges from 16% to 48%, with a higher incidence among elderly patients (Hayashi et al., 2017; McQuade et al., 2016b). In this situation, cancer patients endured the pain from the tumor, but also suffered from long-term constipation until the end of treatment. Severe constipation can even lead to intestinal obstruction and perforation, which can harm the patient’s quality of life (Pehlivan and Nural, 2022; Serrano Falcón et al., 2016). The drugs causing CIC reported in the previous studies were mainly Vinca alkaloids (Vincristine), alkylation reagents (Cyclophosphamide) and platinum-based drugs (Cisplatin, Oxaliplatin) (Escalante et al., 2017; McQuade et al., 2016b; Stojanovska et al., 2014). The current clinical management of CIC mainly relied on the treatment of constipation symptoms, such as osmotic laxatives and enterokinetic drugs (Costilla and Foxx-Orenstein, 2014; Leung et al., 2011; Włodarczyk et al., 2021). However, these treatments are of limited efficacy because of their adverse effects. There is an urgent need to develop new therapeutic drugs to provide new options for clinical treatment (McQuade et al., 2016b; Xue et al., 2011). The pathophysiology of CIC is still unclear, and some studies suggest that chemotherapy drugs generally have significant neurotoxicity, which can affect the smooth muscle of the gastrointestinal tract and damage normal colonic motility, resulting in insufficient intestinal fluid, obstruction of intestinal peristalsis, and ultimately constipation (Escalante et al., 2017). The enteric nervous system can control the absorption, secretion, and motility functions of the intestine. Recent studies have shown that cisplatin can cause morphological and functional alterations in the enteric neurons in a dose-dependent manner. Partial loss of enteric neurons and gial cells was suggested to be responsible for reduced gut motility (Carbone et al., 2016). Li et al. (2020) discovered that cisplatin induces a decrease in interstitial cells of Cajal and alters their morphology within the rat gastrointestinal tract, resulting in gastrointestinal motility disorders. Zou et al. (2022) revealed that mice with cisplatin-induced intestinal injury exhibited a reduction in colonic goblet cells, which may lead to reduced mucin secretion and impaired intestinal barrier function (Duan et al., 2023). Vinca alkaloids (Vincristine) could induce constipation through their neurotoxic effects, which may disrupt gastrointestinal motility (Escalante et al., 2017; Vera et al., 2017). Cyclophosphamide could induce gastrointestinal dysmotility through mechanisms involving inflammatory cytokine-driven neuromuscular dysfunction and structural damage to smooth muscle layers (including apoptosis of interstitial cells of Cajal) (Lee and Kang, 2017). Previous research has shown that oxaliplatin treatment alters colonic neuromuscular function in mice, characterized by elevated nNOS-immunoreactive enteric neuron populations and disrupted nitric oxide-mediated neurotransmission, ultimately leading to significant impairment of colonic motility (McQuade et al., 2016a). In recent study, probiotics could alleviate gastrointestinal complications such as constipation and regulate intestinal microbiota imbalance in cancer patients after chemotherapy (Huang et al., 2023), which indicated that the imbalance of gut microbiota may be another risk factor for CIC.

The gut microbiota, a population of microorganisms residing within the intestinal tract, has been considered as a pivotal factor contributing to the regulation of host health (de Vos et al., 2022). Numerous studies have now established a correlation between imbalances in the gut microbiota and several diseases, including obesity, type 2 diabetes, inflammatory bowel disease, and various types of cancers (Depommier et al., 2019; Lloyd-Price et al., 2019; Wu et al., 2020). The disruption of the gut microbiota may lead to damage to the intestinal barrier and the immune system, thereby triggering various gastrointestinal diseases. By using 16S rRNA microbial genomics and non-target metabolome based on liquid chromatography-mass spectrometry to analyze the gut microbiota composition and serum metabolic profiles of function constipation patients and healthy individuals, the research results of Wang et al. (2023) indicated that there was an imbalance in the gut microbiota of individuals with functional constipation, and constipation in these individuals might be associated with an increase in the *Bacteroidetes* and a downregulation of host arginine biosynthesis upstream metabolites. Zhou et al. (2022) demonstrated that Xiao Chengqi Formula could promote the colonization of *Roseburia* spp., increased the production of the metabolite butyl aminobenzene to alleviate slow transit constipation through 16S rDNA sequencing, metabolomics sequencing, and tissue RNA sequencing. Cisplatin was reported to induce significant changes in the repertoire of intestinal commensal bacteria and restoration of the microbiota through fecal-pellet gavage drives healing of cisplatin-induced intestinal damage (Perales-Puchalt et al., 2018).

Deglycosylated azithromycin (Deg-AZM), a unique metabolite of azithromycin (AZM), has been approved for clinical trials in 2024 as a newly developed Class I drug with good therapeutic effects on chronic constipation. Our previous study found that Deg-AZM had a strong function of promoting intestinal peristalsis (Zhong et al., 2020), Deg-AZM could combine with transgelin target in intestinal smooth muscle cells to promote actin filament coagulation into bundles, thereby enhancing the contractility of smooth muscle cells. Whether Deg-AZM can improve CIC has attracted our attention.

In this study, we aimed to investigate the alleviating effect of Deg-AZM on mice with cisplatin-evoked constipation and the potential microbiota regulation mechanism of Deg-AZM. The diversity and richness of intestinal flora were characterized by 16S rRNA gene sequencing technology, and the metabolic profile changes of related metabolites were analyzed based on untargeted metabolomics technology. Pearson correlation analysis was performed to discuss the association between gut microbiota genome and metabolites.

## 2 Materials and methods

### 2.1 Experimental reagents

Cisplatin was purchased from the MedChemExpress (NJ, USA). Prucalopride Succinate tablets were obtained from Jiangsu Hansoh Pharmaceutical Group Co., Ltd (Jiangsu, China). Lactulose was purchased from Hanmi Pharm. Co. Ltd (Beijing, China). Deg-AZM was synthesized by Porton Pharma Solutions Ltd (Chongqing, China). Powdered activated carbon was provided by Shanghai Yien Chemical Technology Co., Ltd (Shanghai, China). Gum arabic was obtained from Dalian Meilun Biology Technology Co., Ltd (Dalian, China). Carboxymethylcellulose sodium (CMC-Na) was purchased Anhui Sunhere Pharmaceutical Excipients Co., Ltd (Anhui, China).

### 2.2 Experimental animals

A total of 270 healthy male Kunming mice weighing 18–22 g were obtained from SPF (Beijing) Biotechnology Co., Ltd. All mice were provided with standard irradiated food and water under a strict light-dark cycle (12/12 h) at a temperature of 23 ± 2°C and a relative humidity of 50 ± 10%. In the period of modeling and drug treatment, the feces of mice were collected every 4 h to calculate the mass of wet feces, the mass of dry feces, stool water content and particle count of mouse feces. These indicators were employed to evaluate the constipation severity and the progression and recovery from constipation continuously. The animal experiment conducted in this study was approved by the Animal Ethics Committee of Nankai University (Permit No. SYXK 2019–0001) in accordance with the guidelines and recommendations for the Care and Use of Laboratory Animals. The animals were fed adaptively for a week before the formal experiment began.

### 2.3 CIC mice model establishment

Cisplatin powder was accurately weighed and dissolved into 50 mL of 0.9% NaCl solution to prepare cisplatin solution (0.2 mg/mL). Preparation of CIC model using cisplatin as a modeling agent. The constipation induced by cisplatin at different doses was investigated using mouse fecal status as the indicator. Briefly, different doses of cisplatin (1, 1.5, 2, and 3 mg/kg) were administered intraperitoneally to healthy mice, after 12 h of modeling, the overnight feces of the CIC mice were collected and compared with healthy mice.

### 2.4 Deg-AZM treatment on CIC mice model

CIC mice were randomly divided into 7 groups (12 mice in each group), including cisplatin model group (Cis), prucalopride group (Pru), lactulose group (Lac), and Deg-AZM group at doses of 1, 3, 5 and 10 mg/kg, respectively. Healthy mice were selected as the control group (CTL). After 12 h of modeling, the mice in Pru group were intragastrically administered with 0.3 mg/kg of prucalopride as a positive control, the mice in Lac group were intragastrically administered with 1,334 mg/kg of lactulose solution as another positive control, and the mice in Deg-AZM group at different doses were intragastrically administered with 1, 3, 5, and 10 mg/kg of Deg-AZM, respectively, to investigate the therapeutic effect of the Deg-AZM.

### 2.5 Fecal evaluation index

**FIGURE 1:**
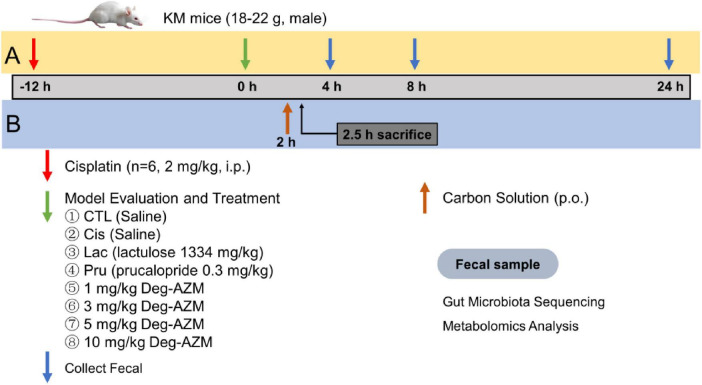
The timeline of animal experiment scheme. **(A)** Fecal evaluation **(B)** Carbon powder propulsion.

Evaluate the therapeutic effect of Deg-AZM on CIC mice model using the mass of wet feces and dry feces, stool water content and particle count of mouse feces as indicators. The timeline of the experimental scheme was shown in Figure 1A. The mice feces were collected after 12 h of modeling to investigate if the model has been successfully prepared. The feces of 0–4 h, 0–8 h and 0–24 h after administration was collected to investigate the therapeutic effect of Deg-AZM on CIC mice. The mass of wet feces was obtained by weighing the fresh feces in every group. The fresh feces were put into an air drying oven at 60°C for 12 h to obtain the mass of dry feces. The number of fecal particles is obtained through visual observation.

Stool water content (%) = (the mass of wet feces–the mass of dry feces)/the mass of wet feces × 100%.

### 2.6 Carbon powder propulsion experiment

The timeline of the experimental scheme was shown in Figure 1B. 2 h after Deg-AZM administration, mice in each group were given a 10 mL/kg dose of 5% activated carbon-10% arabic gum suspension by gavage. After another half hour, the mice were euthanized after cervical spondylolysis. The whole intestine from stomach to anus was taken, and the distance from pylorus to the front end of charcoal powder and from pylorus to ileocecal part was measured to calculate the charcoal powder propulsion rate.

Carbon terminal propulsion rate (%) = distance from pylorus to the front end of carbon powder (cm)/distance from pylorus to ileocecal part (cm) × 100%

### 2.7 16S rRNA gene sequencing and analysis

After 4 h of oral administration of Deg AZM, the fecal samples of mice were collected from the control group (CTL), 2 mg/kg cisplatin group (Model), and 10 mg/kg Deg-AZM group (Treatment), and stored at −80°C. The genome DNA was isolated from the fecal samples using the CTAB method, and its purity and concentration were determined by running it on a 1% agarose gels. Subsequently, primers 515F (5′-GTGCCAGCMGCCGCGGTAA-3′) and 806R (5′-GGACTACHVGGGTWTCTAAT-3′) with barcode were employed to amplify the V4 region of the bacterial 16S rRNA gene. The PCR amplification products were purified, quantified, and normalized to form a sequencing library. The library quality was assessed on the Agilent 5400 (Agilent Technologies Co Ltd., USA). Subsequently, the library was sequenced on an Illumina NovaSeq platform and 250 bp paired-end reads were generated.

The raw sequences obtained from sequencing were imported into QIIME2 for further processing, and operational taxonomic units (OTUs) were obtained through clustering based on 97% sequence similarity. The taxonomic classification of the obtained OTUs was assessed using the RDP Classifier algorithm, applying a confidence threshold of 70%, against the Silva 16S rRNA database. The core-diversity plugin within QIIME2 was employed to calculate both α diversity and β diversity. LEfSe analysis was used to detect significant distinctions in species classification levels across various groups.

### 2.8 LC-MS based metabolomics analysis of fecal samples

The fecal sample (100 mg) was ground in liquid nitrogen with 500 μL 80% methanol-water (*v/v*) to obtain a uniform solution. After vortex-mixing and centrifuging at 15,000 *g* for 20 min at 4°C, the supernatant was collected from the homogeneous solution and diluted with water until the methanol content reached 53%, then centrifuge the diluent again under the same conditions, the supernatant was collected analyzed on LC-MS system subsequently. In addition, equal volumes sample from each experimental specimen were mixed to obtain QC samples.

The analysis was conducted on Vanquish UHPLC and Q Exactive HF-X (Thermo Fisher). Chromatographic separation process was carried out on a Hypesil Gold column (100 × 2.1 mm, 1.9 μm). The flowrate was 0.2 mL/min with column temperature at 40°C. Elution was accomplished using mobile phase of water containing 0.1% formic acid (A) and methanol (B) by a gradient elution program as follows: 98% (A) from 0 to 1.5 min, 98%–15% (A) from 1.5 to 3 min, 15%–0% (A) from 3 to 10 min, 0%–98% (A) from 10.1 to 12 min. The ESI source set as follows: spray voltage 3.5 KV, sheath gas flow rate 35 psi, aux gas flow rate 10 L/min, capillary temperature 320°C, aux gas heater temperature 350°C. The mass range was from m/z 100 to 1,500. MS/MS secondary scanning was data-dependent scans.

### 2.9 Data statistics methods

GraphPad Prism software (GraphPad, San Diego, CA, USA) was adopted to process the experimental data, which were presented as the mean ± standard deviation. The Variance homogeneity was analyzed using F test. Unpaired T test was used to compare data between different groups, statistical significance was considered as a *p*-value less than 0.05.

LC/MS raw data was imported into Compute Discover 3.1 software for peak recognition, peak alignment, and normalization. Metabolite identification and annotation were combined with human metabolome database (HMDB), Kyoto Encyclopedia of Genes and Genomes database (KEGG), Lipid metabolites and pathways strategy database (LIPID MAPS). Multivariate analysis and differential metabolite screening were performed on SMICA software, with the selection criteria set as VIP > 1, Log_2_(FC) > 1, and *p* < 0.05. Differential metabolites were input into MetaboAnalyst 5.0 platform for pathway enrichment and topology analysis.

Pearson correlation analysis was performed between altered metabolites and perturbed top 20 gut microbiota genus screened. Positive correlation was displayed in red, while negative correlation was displayed in blue.

## 3 Results

### 3.1 CIC model evaluation

**FIGURE 2:**
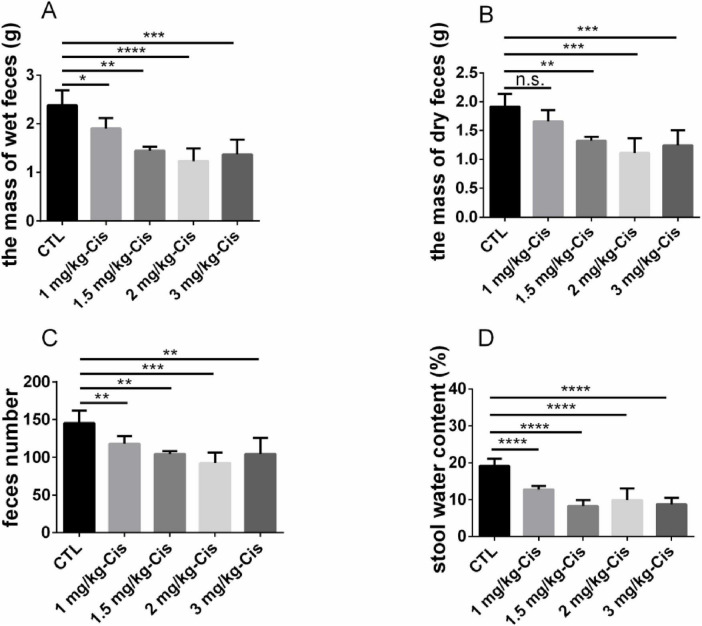
Evaluation of constipation models induced by different doses of cisplatin. **(A)** the mass of wet feces in 0–12 h, **(B)** the mass of dry feces in 0–12 h, **(C)** feces number in 0–12 h, **(D)** stool water content in 0–12 h. Data were presented as the mean ± SD, *n* = 6. *p < 0.05; **p < 0.01; ***p < 0.001; ****p < 0.0001 compared to the CTL group. All *P*-values in F test to compare variances were greater than 0.05.

The mass of wet feces and dry feces was shown in Figure 2, and number of feces in mice after 12 h administration of different doses of cisplatin. Compared with the control group (CTL), the mass of wet feces and number of feces in mice were significantly reduced after inducing with cisplatin at different doses (*p* < 0.01). Except for 1 mg/kg, cisplatin at the dosage of 1.5, 2 and 3 mg/kg could significantly reduce the mass of dry feces in mice (*p* < 0.01). The reduction of the mass of wet feces and dry feces, and number of feces in mice induced by cisplatin was related to its dosage. However, mice showed signs of mental fatigue and hair loss after administration of 3 mg/kg cisplatin. In the subsequent experiments, 2 mg/kg cisplatin was used to induce CIC models.

### 3.2 Pharmacodynamic evaluation of Deg-AZM

**FIGURE 3:**
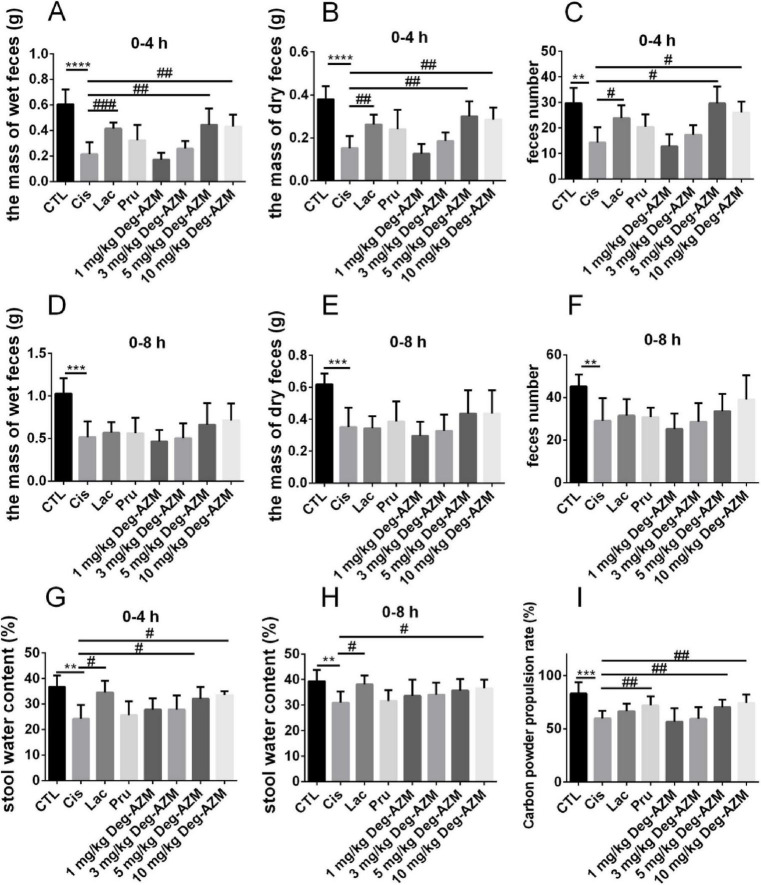
Pharmacodynamic evaluation of Deg-AZM in relieving cisplatin-evoked constipation model. **(A)** The mass of wet feces in 0–4 h, **(B)** the mass of dry feces in 0–4 h, **(C)** feces number in 0–4 h, **(D)** the mass of wet feces in 0–8 h, **(E)** the mass of dry feces in 0–8 h, **(F)** feces number in 0–8 h, **(G)** stool water content in 0–4 h, **(H)** stool water content in 0–8 h, **(I)** Carbon powder propulsion rate. Data were presented as the mean ± SD, *n* = 6. **p < 0.01; ***p < 0.001; ****p < 0.0001 compared to the CTL group. #*p* < 0.05; ##*p* < 0.01; ###*p* < 0.001 compared to the Cis group. All *P*-values in F test to compare variances were greater than 0.05.

The efficacy of Deg-AZM at different dose was evaluated on the established CIC model. Figures 3A–F showed the mass of wet feces and dry feces, and number of mice feces from each group within 4 h and 8 h after administration, with corresponding 24 h data in Supplementary Figure 1. Compared with the CTL group, the mass of wet feces and dry feces, and number of mice feces in the Cis group were significantly reduced (*p* < 0.01), indicating that the experimental mice were in a state of constipation. Within 4 h after administration, compared with the Cis group, the mass of wet feces and dry feces, and number of mice feces in the Lac group were significantly increased (*p* < 0.05), while there were no significant changes in the Pru group. It indicated that lactulose could alleviate fecal index in CIC mice, but prucalopride has a poor effect on CIC. Therefore, lactulose was used as a positive control in this experiment. In addition, within 4 h after administration of Deg-AZM, the mass of wet feces and dry feces, and number of mice feces in the Deg-AZM group increased with the increase of dosage. Among them, the mass of wet feces and dry feces, and number of mice feces were significantly higher than those in the Cis group after administration of 5 mg/kg and 10 mg/kg of Deg-AZM (*p* < 0.05), and the effect was comparable to that of the Lac group, indicating that Deg-AZM could dose-dependent alleviate constipation symptoms in CIC mice, with an effective dose of 5 mg/kg. However, within 8 h after administration, the constipation symptoms in CIC mice were alleviated, and the positive drug and Deg-AZM did not show any therapeutic effect. Figures 3G, H, respectively, illustrated the stool water content at 0–4 h and 0–8 h. Compared to the CTL group, the stool water content in the Cis group was significantly reduced at 4 h and 8 h post-administration, indicating that the mice in the Cis group were in a state of constipation. The stool water content in the Lac group was significantly higher than that in the Cis group, demonstrating that lactulose has a better ability to increase stool water content. At the same time, compared to the Cis group, Deg-AZM at a dosage of 10 mg/kg also significantly increased stool water content (*p* < 0.05).

By comparing the intestinal carbon powder propulsion rates in mice of different groups, we evaluated the effect of Deg-AZM on the intestinal motility function of CIC model mice. As shown in Figure 3I, compared to the CTL group (83.1 ± 10.4%), the intestinal carbon powder propulsion rate of the Cis group (59.8 ± 7.2%) was significantly reduced, indicating slowed intestinal movement in CIC mice, which was also a manifestation of constipation. Compared to the Cis group, the carbon powder propulsion rate of mice in the Pru group (72.1 ± 8.4%) significantly increased (*p* < 0.05), while the Lac group showed no significant change. Therefore, prucalopride was used as the positive drug in this experiment. Additionally, the carbon powder propulsion rate of mice after Deg-AZM administration was positively correlated with its dosage. Specifically, the carbon powder propulsion rates of mice were 70.4 ± 7.0% and 74.4 ± 8.1%, respectively, when Deg-AZM was administered at dosages of 5 mg/kg and 10 mg/kg, which were significantly higher than those of the mice in the Cis group and showed similar effects to the Pru group.

### 3.3 Deg-AZM regulates the gut microbiota of mice with cisplatin-evoked constipation

**FIGURE 4:**
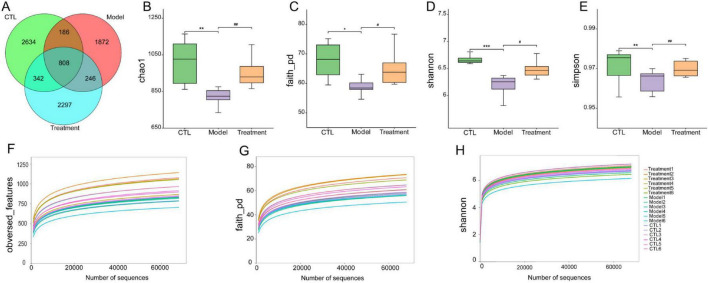
Effects of Deg-AZM treatment on the gut microbiota in mice. **(A)** Venn diagram of OTUs, **(B)** Chao1 index, **(C)** Faith_pd index, **(D)** Shannon index, **(E)** Simpson index, **(F)** Rarefaction curve of obversed_features, **(G)** Rarefaction curve of faith_pd, **(H)** Rarefaction curve of Shannon. Data were presented as the mean ± SD, *n* = 6. *p < 0.05; **p < 0.01; ***p < 0.001 compared to the CTL group. #*p* < 0.05; ##*p* < 0.01 compared to the Model group. All *P*-values in F test to compare variances were greater than 0.05.

Sequencing of the 16S rRNA was conducted on 18 samples from three different groups to assess the differences in the structure and abundance of the gut microbiota among the groups. After undergoing preliminary processing and filtration, a total of 1569010 sequence reads were obtained from the raw sequencing data. Subsequently, sequence clustering analysis was performed with a 97% similarity threshold, resulting in the identification of 8,358 operational taxonomic units (OTUs). The OTUs in the CTL group, Model group, and Treatment group were 2,634, 1,872, and 2,297, respectively, indicating a decrease in gut microbiota after cisplatin modeling (Figure 4A). Deg-AZM treatment could alleviate the disturbance of gut microbiota and basically restore to normal levels.

**FIGURE 5:**
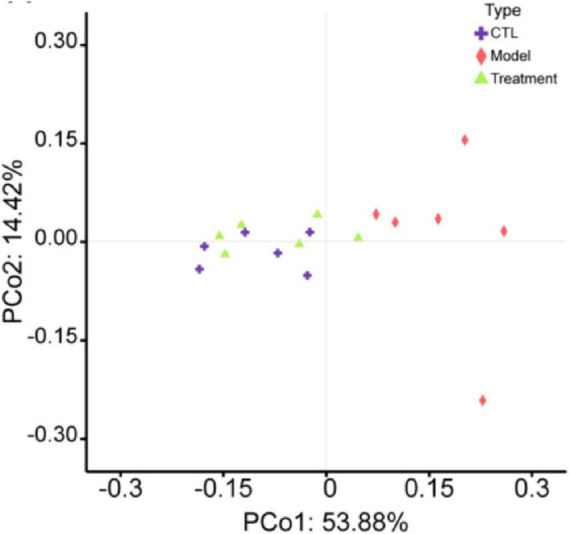
Principal coordinates analysis (PCoA) of the gut microbiota β-diversity in mice.

The α-diversity of the gut microbiota can reflect the diversity and richness of microbial communities in the samples. Chao1, Faith_pd, Shannon, and Simpson indexes are indicators used to characterize the α-diversity of communities. The Chao1 (*p* < 0.01), Faith_pd (*p* < 0.05), Shannon (*p* < 0.001), and Simpson (*p* < 0.01) indexes in the model group were significantly lower than those in the CTL group (Figures 4B–E), indicating a notable decrease in community diversity and richness in this group. The sample rarefaction curve could reflect whether the sequencing data amount is reasonable. As the sequencing depth continues to increase, the curve tended to flatten, indicating that the sequencing data amount of this experimental sample is sufficient (Figures 4F–H). β-diversity refers to the comparison of differences in community structure between different groups. In the principal coordinates analysis (PCoA) analysis chart (Figure 5), the contribution rates of the first principal coordinates (PCo 1) and the second principal coordinates (PCo 2) to the variation were 53.88% and 14.42%, respectively. Compared with CTL group, the gut microbiota of the Model group deviated from the baseline structure and showed significant deviation along the positive direction of the PCo 1. The difference between the Treatment group and the CTL group was not significant. The aforementioned results indicate that the gut microbiota structure of mice was altered following cisplatin injection. Treatment with Deg-AZM was found to restore the gut microbiota composition, making it more similar to that of the control group in terms of species composition.

**FIGURE 6:**
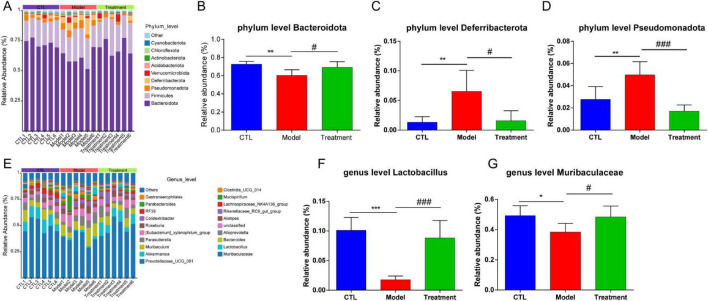
Analysis of gut microbiota composition of each sample. **(A)** Relative abundance of the gut microbiota at the phylum level, **(B)** Comparison of *Bacteroidota* relative abundance, **(C)** Comparison of *Deferribacterota* relative abundance, **(D)** Comparison of *Pseudomonadota* relative abundance, **(E)** Relative abundance of the gut microbiota at the genus level, **(F)** Comparison of *Lactobacillus* relative abundance, **(G)** Comparison of *Muribaculaceae* relative abundance. Data were presented as the mean ± SD, *n* = 6. *p < 0.05; **p < 0.01; ***p < 0.001 compared to the CTL group. #*p* < 0.05; ###*p* < 0.001 compared to the Model group. All *P*-values in F test to compare variances were greater than 0.05.

Based on the absolute abundance and annotation information of OTUs, the gut microbiota of each group was analyzed at the phylum and genus levels to identify differential gut microbiota. Figures 6A–D presented the differences in microbial composition at the phylum level among the groups, indicating that the *Bacteroidota*, *Firmicutes*, *Pseudomonadota* and *Deferribacterota* were the predominant microbial in each group (Figure 6A). Specifically, compared to the CTL group, the relative abundance of *Bacteroidota* in the Model group significantly decreased (*p* < 0.01), while the relative abundance of *Deferribacterota* and *Pseudomonadota* significantly increased (*p* < 0.01, Figures 6B–D). However, compared to the Model group, the relative abundance of *Bacteroidota* in the Treatment group significantly increased (*p* < 0.05), while the relative abundance of *Deferribacterota* and *Pseudomonadota* significantly decreased (*p* < 0.05). Figures 6E–G showed the differences in microbial composition at the genus level among the groups, indicating that levels of *Lactobacillus* and *Muribaculaceae* in the Model group significantly decreased compared to the CTL group (*p* < 0.05). In comparison to the Model group, the Treatment group showed a significant increase in the relative abundance of *Lactobacillus* and *Muribaculaceae* (*p* < 0.05). Overall, Deg-AZM effectively restored the imbalance of *Bacteroidota*, *Deferribacteres*, and *Pseudomonadota* in the gut microbiota induced by cisplatin at the phylum level. Meanwhile, at the genus level, it positively impacted the abundance of *Lactobacillus* and *Muribaculaceae*, thereby contributing to a balanced and healthy gut microbial community. This could potentially provide a new mechanistic explanation for Deg-AZM treatment of CIC. Previous studies have indicated that cisplatin can lead to structural imbalance of gut microbiota, increasing the abundance of *Deferribacteres* and *Pseudomonadota* while decreasing the abundance of *Bacteroides* (Wu et al., 2019), which was consistent with our results.

**FIGURE 7:**
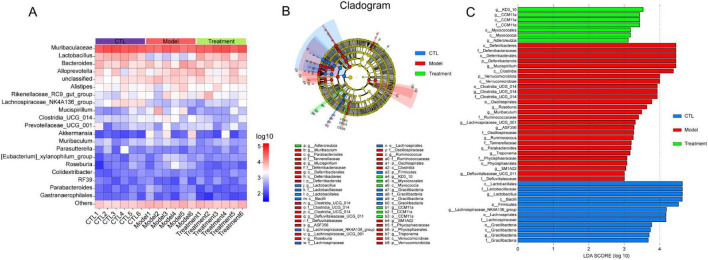
Analysis of different gut microbiota between groups. **(A)** Relative abundance of the gut microbiota at genus level heatmap, **(B)** Cladogram of differentially abundant microbiota, **(C)** LDA score plot.

To visually display the differences in gut microbiota among different groups, Figure 7A showed a heatmap of the top 20 microbiota at the genus level. It can be observed that compared to the CTL group, *Lactobacillus* and *Lachnospiraceae_NK4A136_group* in the Model group were significantly reduced, and Deg-AZM significantly increased the levels of *Lactobacillus*. Furthermore, linear discriminant analysis effect size (LEfSe) analysis was used to identify different microbiota between groups. The cladogram (Figure 7B) illustrated the taxonomic hierarchy of species with significant differences between groups, while the LDA score histogram (Figure 7C) displayed significantly enriched species within each group. In the CTL group, the abundance of *o_Lactobacillales*, *f_Lactobacillaceae*, *g_Lactobacillus*, and *g_Lachnospiraceae_NK4A136_group* was higher, whereas the Model group exhibited higher abundance of *c_Deferribacteres*, *f_Deferribacteraceae*, *g_Mucispirillum*, and *c_Clostridia*. In the treatment group, the proportions of *g_KD3_10*, *g_CCM11a* and *o_Myxococcales* were relatively higher. This indicated significant changes in the gut microbiota of CIC mice, which could be restored to near levels of CTL group after treatment of Deg-AZM.

### 3.4 Metabolomics analysis on treatment effects of Deg-AZM.

Untargeted metabolomics techniques were employed in the study, and 782 metabolites in positive ion mode and 432 metabolites in negative ion mode were identified among 18 samples. The data of positive and negative ions were combined to analyze the metabolite profile of 1214 kinds of metabolites.

**FIGURE 8:**
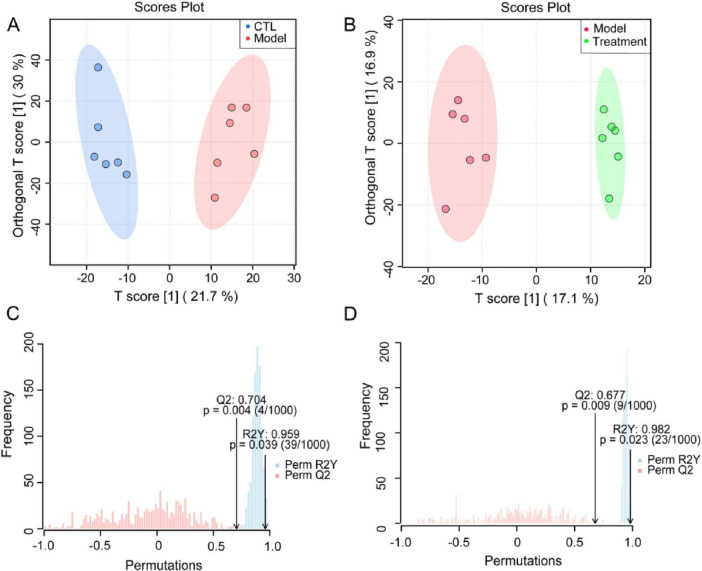
Supervised discriminant analysis of fecal metabolome. OPLS-DA score plot of **(A)** CTL vs. Model, **(B)** Treatment vs. Model. OPLS-DA model assessment of **(C)** CTL vs. Model, **(D)** Treatment vs. Model.

An orthogonal partial least squares discriminant analysis (OPLS-DA) model was established to compare the composition and structure of metabolites between different groups. OPLS-DA analysis showed that there were significant differences in fecal metabolites between the cisplatin induced Model group and the CTL group, as well as between the Treatment group and the Model group (Figures 8A, B), indicating that cisplatin modeling and Deg-AZM treatment could lead to changes in metabolite profile of mice. The quality assessment of the model was conducted through 1,000 permutation tests (Figures 8C, D), which indicated effective predictive ability (Q2 = 0.704, Q2 = 0.677, *p* < 0.01) and significant explanatory power (R2Y = 0.959, R2Y = 0.982, *p* < 0.05), along with an acceptable fit of the OPLS-DA model.

**FIGURE 9:**
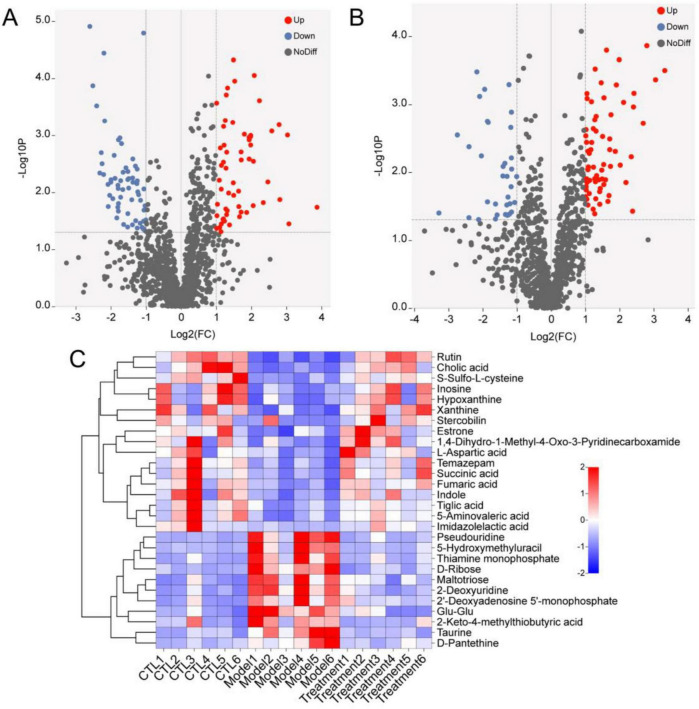
Differential metabolites analysis. Volcano map of differential metabolites of **(A)** CTL vs. Model, **(B)** Treatment vs. Model. **(C)** Heat map of 28 differential metabolites.

We used the volcano plot to identify the key metabolite characteristics that distinguish different groups (Figures 9a–b). Compared to the CTL group, Model group increased 58 metabolites and decreased 62 metabolites; Compared to the Model group, 67 metabolites were up-regulated and 34 metabolites were down regulated after treatment with Deg-AZM. The above metabolites were intersected between the groups, 28 metabolites met the criteria of VIP > 1, *p* < 0.05 and log2| (FC) | > 1 (FC: fold change), and were finally identified as potential biomarkers (Table 1). In order to visualize and characterize the differential metabolites among these three groups effectively, we standardized the contents of the 28 different metabolites and constructed the heat map (Figure 9C).

**TABLE 1:** Some differential metabolites in mice feces after treatment.

Metabolite	Formula	m/z	Model/CTL		Treatment/model	
			VIP	FC	VI	FC
5-Hydroxymethyl uracil	C_5_H_6_N_2_O_3_	143.05	1.84	8.21	1.83	0.15
Thiamine monophosphate	C_12_H_17_N_4_O_4_PS	345.08	1.85	6.95	1.76	0.24
2′-Deoxyadenosine 5′-monophosphate	C_10_H_14_N_5_O_6_P	330.06	1.80	6.02	1.42	0.43
Pseudouridine	C_9_H_12_N_2_O_6_	243.06	1.97	4.72	1.89	0.27
Maltotriose	C_18_H_32_O_16_	505.18	1.81	3.96	1.41	0.45
Taurine	C_2_H_7_NO_3_S	126.02	1.76	3.85	1.90	0.28
D-Pantethine	C_22_H_42_N_4_O_8_S_2_	555.25	1.63	3.13	1.40	0.41
2-Deoxyuridine	C_9_H_12_N_2_O_5_	229.08	1.94	2.89	1.91	0.45
Glu-Glu	C_10_H_16_N_2_O_7_	275.09	1.97	2.82	1.86	0.44
D-Ribose	C_5_H_10_O_5_	149.05	2.01	2.45	2.02	0.43
2-Keto-4-methylthiobutyric acid	C_5_H_8_O_3_S	147.01	1.32	2.31	1.70	0.39
Stercobilin	C_33_H_46_N_4_O_6_	595.35	1.30	0.48	1.63	2.63
1,4-Dihydro-1-Methyl-4-Oxo-3-Pyridinecarboxamide	C_7_H_8_N_2_O_2_	153.07	1.62	0.48	1.86	2.48
L-Aspartic acid	C_4_H_7_NO_4_	134.04	1.57	0.48	1.65	2.81
Xanthine	C_10_H_12_N_4_O_6_	153.04	1.45	0.43	2.04	2.44
Indole	C_8_H_7_N	118.07	1.77	0.42	1.86	2.03
Temazepam	C_16_H_13_ClN_2_O_2_	301.08	1.84	0.41	1.80	2.24
Estrone	C_18_H_22_O_2_	539.32	1.37	0.38	1.55	3.23
Fumaric acid	C_4_H_4_O_4_	115.00	1.84	0.35	1.93	2.42
Imidazolelactic acid	C_6_H_8_N_2_O_3_	109.04	1.80	0.34	1.94	2.07
5-Aminovaleric acid	C_5_H_11_NO_2_	118.09	1.75	0.32	1.81	2.27
Tiglic acid	C_5_H_8_O_2_	101.06	1.66	0.32	1.68	2.11
Succinic acid	C_4_H_6_O_4_	117.02	1.78	0.30	1.81	3.28
Hypoxanthine	C_5_H_4_N_4_O	137.05	1.41	0.29	1.62	3.01
Inosine	C_10_H_12_N_4_O_5_	269.09	1.39	0.24	1.49	3.16
S-Sulfo-L-cysteine	C_3_H_7_NO_5_S_2_	199.97	1.92	0.22	2.09	3.07
Cholic acid	C_24_H_40_O_5_	407.28	1.88	0.19	2.05	3.97
Rutin	C_27_H_30_O_16_	609.14	1.97	0.18	1.98	5.35

**FIGURE 10:**
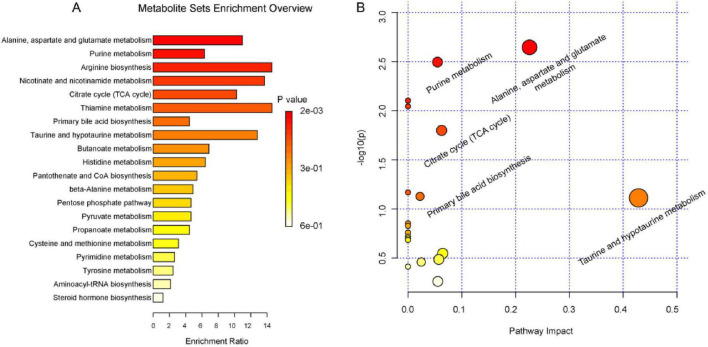
Summary of pathway analysis. **(A)** pathway enrichment analysis. **(B)** pathway topology analysis.

Metaboanalyst 5.0 performed enrichment analysis and topology analysis of metabolic pathways. The results showed that Deg-AZM alleviated the symptoms of cisplatin-evoked constipation in mice by regulating a variety of metabolic pathways, including Alanine, aspartate and glutamate metabolism, Purine metabolism, Citrate cycle (TCA cycle), Taurine and hypotaurine metabolism, Cysteine and methionine metabolism, Pyrimidine metabolism, Tyrosine metabolism, Steroid hormone biosynthesis (Figure 10). Among the metabolites involved in the above metabolic pathways, L-aspartic acid, succinic acid, fumaric acid, inosine, hypoxanthine and xanthine were significantly decreased in the Model group, while pseudouridine, 2-deoxyuridine and taurine were significantly increased in the Model group. These indicated that cisplatin induced changes in the content of metabolites in organisms, thereby damaging the normal intestinal function, and Deg-AZM treatment reversed the changes of these metabolites.

Pearson correlation analysis was used to explore the potential associations between intestinal flora and fecal biomarkers (Supplementary Figure 2), which showed that *Lactobacillus* was significantly positively correlated with inosine, hypoxanthine, cholic acid, xanthine, L-aspartic acid, succinic acid, fumaric acid, and negatively correlated with pseudouridine, D-ribose, taurine, and 5-hydroxymethyl uracil. *Muribaculaceae* was positively correlated with inosine, cholic acid, xanthine, succinic acid, fumaric acid, and negatively correlated with taurine. *Lachnospiraceae_NK4A136_group* was positive correlation with inosine and cholic acid.

## 4 Discussion

CIC has become an urgent problem that needs to be addressed as it further exacerbates the quality of life for cancer patients. Drugs that often cause constipation include vincristine, vinblastine, oxaliplatin, cisplatin, and thalidomide (Escalante et al., 2017; McMillan et al., 2013; McQuade et al., 2016b). This study established a CIC model by intraperitoneal injection of cisplatin into mice. Limited researches existed on cisplatin- evoked constipation in mice. While some studies indicate that a 5 mg/kg cisplatin dose can significantly delay gastric emptying and induce acute gastrointestinal effects (Shahid et al., 2018). Our study found that administration of 5 mg/kg cisplatin led to a subset of mice mortality. In contrast, when the dosage was adjusted to 1, 1.5, 2 or 3 mg/kg, the mice survived and exhibited constipation with decreasing of stool water content significantly. Based on this model, we completed the pharmacodynamic evaluation of Deg-AZM and explored its mechanism of treating CIC by 16S rRNA sequencing and metabonomic analysis.

Currently, there are no targeted therapeutic drugs for CIC in clinical practice. Traditional treatments for CIC often include bulk-forming laxatives, osmotic laxatives, and stimulant laxatives. However, these therapies often exhibit various side effects, delayed onset of action, and risks of electrolyte imbalances or long-term dependency (Costilla and Foxx-Orenstein, 2014; Leung et al., 2011; McQuade et al., 2016b; Włodarczyk et al., 2021). In this study, prucalopride and lactulose were selected as positive controls in this study to comprehensively evaluate the preclinical efficacy of Deg-AZM. Prucalopride improved intestinal motility in cisplatin-induced constipated mice but showed no significant effect on fecal indicators. Additionally, as a 5-HT4 receptor agonist, prucalopride may carry cardiovascular side effects in clinical use. Lactulose, a commonly prescribed agent for CIC patients, significantly improved fecal metrics in the cisplatin-induced constipation model but failed to enhance intestinal motility, lactulose is associated with adverse effects such as bloating, diarrhea, vomiting, and electrolyte imbalances clinically. In contrast, Deg-AZM restored both intestinal motility and fecal parameters in cisplatin-treated mice within 4 h of administration, demonstrating comparable efficacy to prucalopride and lactulose but without observed side effects such as arrhythmia, diarrhea, or vomiting during the trial. Notably, unlike conventional laxatives that directly stimulate secretion or excretion, Deg-AZM’s microbiota-modulating capability may address the root cause of chemotherapy-induced dysbiosis, potentially reducing long-term dependency risks. These findings suggested that Deg-AZM could offer a novel therapeutic approach for managing CIC.

Gut microbiota homeostasis is closely related to host health. According to previous studies, cisplatin could lead to gut microbiota imbalance and cause diseases such as colonic mucositis, kidney injury or liver injury (Perales-Puchalt et al., 2018; Wang L. et al., 2022; Zou et al., 2022). Through 16S rRNA sequencing technology, we found that there were significant differences in the diversity and richness of gut microbiota between mice with cisplatin-evoked constipation and healthy mice. Compared with the CTL group, the abundance of *Bacteroidota*, *Lactobacillus*, *Muribaculaceae* and *Lachnospiraceae_NK4A136_group* in the Model group were decreased, while the abundance of *Deferribacterota* and *Pseudomonadota* were increased.

Principal component analysis (PCA) is an unsupervised dimensionality reduction technique, mainly used for exploratory data analysis to help understand the structure of the data and identify the main sources of variation. In contrast, OPLS-DA is a supervised analytical method specifically designed for classification problems, which can better distinguish differences between groups. In the metabolomics analysis of this study, we employed both PCA and PCoA to explore inter-group differences. The results showed that PCA failed to clearly differentiate between groups. In contrast, OPLS-DA effectively highlighted the differences in metabolite profiles between groups. Therefore, we ultimately opted to use OPLS-DA for our analysis. In comparison to the Model group, Deg-AZM alleviated constipation by increasing in the relative abundance of *Lactobacillus* and *Muribaculaceae*. By exploring the potential associations between intestinal flora and fecal biomarkers based on Pearson correlation analysis, we found that *Lactobacillus* was significantly positively correlated with inosine, hypoxanthine, cholic acid, xanthine, L-aspartic acid, succinic acid, fumaric acid. *Lactobacillus* may be the host of the above metabolites or promote its occurrence. Previous studies have reported that *Lactobacillus* contributes to the biosynthesis of the flavonoid compound rutin (Li et al., 2022) and involve in the metabolic process of cholic acid (Ferreira-Lazarte et al., 2021). Additionally, *Muribaculaceae* was found positively correlated with inosine, cholic acid, xanthine, succinic acid, fumaric acid, which was also reported to increase the content of acetate and succinic acid from intestinal metabolites in previous study (Ismaeel et al., 2023). *Muribaculaceae* may be the host of these metabolites or promote its occurrence. Further researches on the relationship among metabolites, *Lactobacillus*, *Muribaculaceae* and constipation is needed.

**FIGURE 11:**
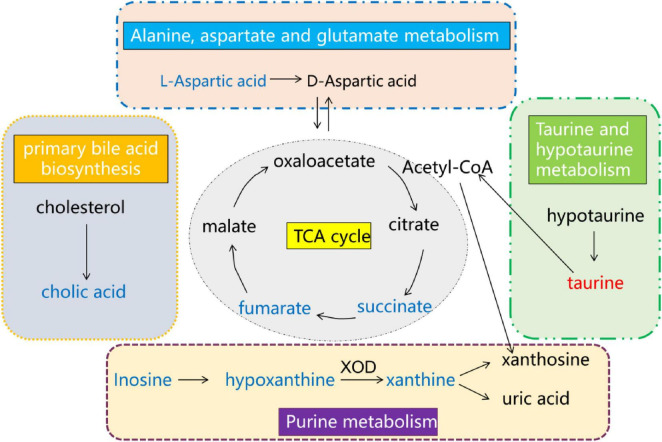
Overall network of pathways involved in Deg-AZM alleviating CIC symptoms in mice analyzed using the metabolomics approach. Compared with the CTL group, red indicates an increase in the content of the Model group, while blue indicates a decrease in the content of the Model group.

Through the metabolomic analysis, we found that there was an obvious separation among cisplatin model group, CTL group and Deg-AZM group. A total of 28 different metabolites were identified among the three groups. Further metabolic pathway analysis identified metabolic pathways that may be related to the regulation of constipation by Deg-AZM. The metabolic pathways depicted in Figure 11 may play a crucial role in alleviating CIC. The changes of amino acid metabolism related pathways accounted for a high proportion of all enriched pathways. Among them, alanine, aspartate and glutamate metabolism were the most significantly altered metabolic pathways. In the Model group, the content of L-aspartic acid involved in this metabolic pathway was significantly reduced, indicating that this metabolic pathway was damaged after cisplatin injection. L-aspartic acid is a non-essential amino acid in mammals, but it participates in a variety of biochemical reactions in the organism and is converted into other amino acids, including arginine, glutamine and glutamate, to exert its beneficial functions (Morris, 2016). A previous study has reported that L-aspartic acid regulates stem cell proliferation and differentiation to reduce epithelial cell damage (Wang D. et al., 2022). Meanwhile, researchers have found that L-aspartic acid can increase gut microbiota α-diversity, improve of gut microbiota abundance and regulate of intestinal immune function (Bin et al., 2017). A recent study showed that zinc L-aspartate can regulate Wnt/β-Catenin pathway and protect the integrity of intestinal mucosa (Zhou et al., 2020).

Citrate cycle (TCA cycle) is an important energy metabolism pathway in organisms, which promotes energy production and the synthesis of other metabolites. Succinic acid and fumaric acid are representative intermediates involved in TCA cycle. In the normal TCA cycle of the body, succinic acid is produced by succinate dehydrogenase, and fumaric acid continues to participate in the subsequent energy metabolism pathway (Connors et al., 2019; Tong et al., 2022). This metabolic process is carried out in the mitochondria of organisms and provides energy for a number of life activities. Energy metabolism disorders can induce constipation because intestinal peristalsis requires a large amount of energy to be provided (Liu et al., 2023). Our study found that the content of fumaric acid and succinic acid decreased significantly after cisplatin injection, indicating that the normal energy metabolism of the body was abnormal. It has been reported that cisplatin can induce mitochondrial damage leading to kidney, lung and intestinal injury (Han et al., 2021; Liu et al., 2022; Zhu et al., 2020). The results of this study may indicate that cisplatin can induce mitochondrial damage leading to abnormal energy metabolism and induce constipation. The treatment of Deg-AZM can adjust the TCA cycle disorder caused by cisplatin and produce the therapeutic effect of alleviating constipation.

Purine metabolism is a process of cellular energy metabolism, in which inosine is transformed into hypoxanthine. Hypoxanthine can be converted to xanthine by xanthine oxidase (XOD), and then to uric acid (Dewulf et al., 2022). In this study, we found that the contents of inosine, xanthine and hypoxanthine in model group were significantly decreased, and Deg-AZM could increase the contents of these metabolites. As a metabolite of intestinal flora, inosine was reported to alleviate colitis and regulate intestinal immunity (Zhu et al., 2022). Previous study showed that hypoxanthine could regulate the energy metabolism of intestinal epithelial cells and repair the intestinal barrier function (Lee et al., 2018), at the same time, hypoxanthine could increase intracellular ATP and improve the polymerization of cytoskeleton G-actin to F-actin, which was closely related to promoting intestinal peristalsis function.

The primary bile acid biosynthesis pathway was also affected by cisplatin, and the cholic acid involved in this pathway is significantly reduced in the model group. It was reported that the primary bile acid biosynthesis in slow transit constipation patients was disordered, and the cholic acid was significantly reduced (Fan et al., 2022). In addition, it was found that taurine could greatly reversed KBrO_3_-induced intestinal tissue damage which was closely related to the chemical protection of oxidative stress in the previous study (Ahmad et al., 2015). In our study, taurine was significantly up-regulated after cisplatin injection, which indicated that taurine content is increased after intestinal damage to reduce the intestinal toxicity of cisplatin.

Deg-AZM reshapes gut microbiota balance by increasing *Bacteroidota*, *Lactobacillus*, and *Muribaculaceae*, while inhibiting *Proteobacteria*. *Bacteroidota* produce SCFAs like acetate and propionate, which enhance gut barrier function by promoting tight junction protein expression, thereby reducing cisplatin-induced intestinal permeability. *Lactobacillus* restoration elevates gut lactate levels, inhibiting pathogenic *Proteobacteria* proliferation and reducing systemic inflammation, which lowers infection risks in chemotherapy patients. *Muribaculaceae* increase may improve gut energy metabolism by promoting metabolites like succinate, supporting intestinal epithelial repair. Metabolomic reprogramming by Deg-AZM restores TCA cycle metabolites (succinate, fumarate), enhancing intestinal epithelial energy supply and gut motility, thereby reducing chemotoxicity. Additionally, reprogramming of bile acid metabolism may accelerate cisplatin detoxification. These changes may indirectly enhance chemotherapy tolerance and improve patient quality of life.

## 5 Conclusion

This study investigated the alleviating effect of Deg-AZM on cisplatin-evoked constipation on mice and the potential microbiota regulation mechanism of Deg-AZM. Firstly, a stable CIC mice model was successfully established by optimizing the modeling dose of the chemotherapy drug cisplatin. The mass of wet feces and dry feces, and feces number in CIC mice were significantly reduced compared to normal mice. Subsequently, Deg-AZM was shown to reduce CIC by increasing the mass of wet feces and dry feces, and feces number in CIC mice, and promoting intestinal motility in CIC mice, with a dose-dependent manner. In addition, the regulation of gut microbiota by Deg-AZM was analyzed through 16S rRNA sequencing, and changes in metabolites were analyzed using untargeted metabolomics. The results showed that Deg-AZM can alleviate cisplatin-evoked constipation in mice by reshaping the structure of gut microbial community, which will hopefully provide a new drug choice for the treatment of constipation in chemotherapy patients.

## Data Availability

The raw sequencing data analyzed in this study was generated by a third-party testing company. Unfortunately, due to their data retention policy, the original sequencing data is not available. However, processed datasets (such as OTU tables and metabolite profiles) and detailed experimental protocols used in this study are retained by the authors and are available upon reasonable request.
