# Supplementary material for: Deglycosylated azithromycin alleviates cisplatin-evoked constipation in mice by altering host metabolome and gut microbiota composition
Source: Front Microbiol. 2025 May 21;16:1437662. doi: 10.3389/fmicb.2025.1437662 (PMC12136492; doi:10.3389/fmicb.2025.1437662)
Supplement: Supplementary file 1 [file Data_Sheet_1.docx]

***1. Fecal evaluation of 0-24h.***


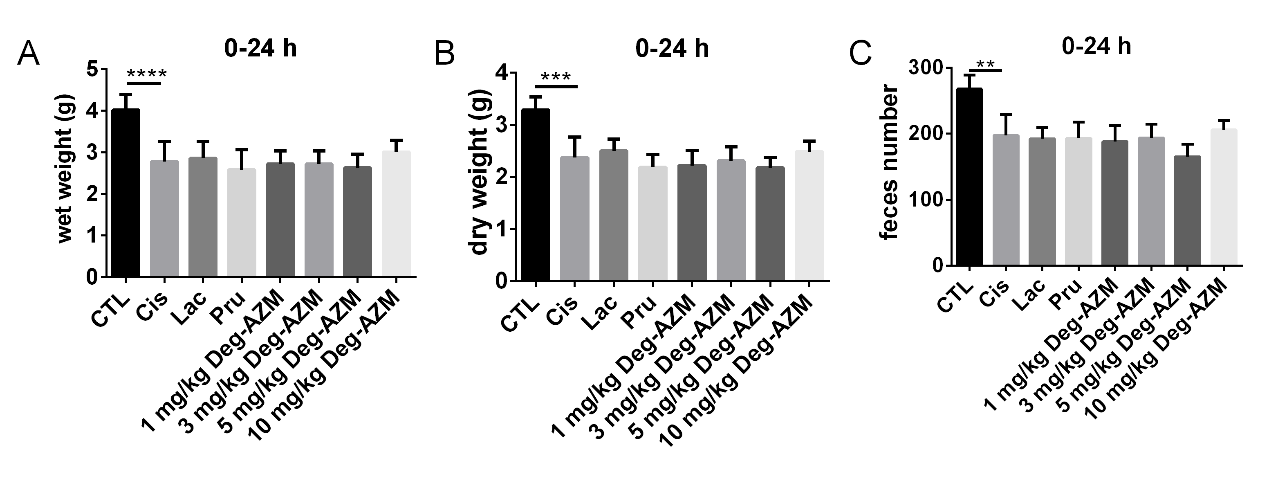


**Figure S1**. Pharmacodynamic evaluation of Deg-AZM in relieving cisplatin induced constipation model. (A) wet weight of feces in 0-24 h, (B) dry weight of feces in 0-24 h, (C) feces number in 0-24 h. ** *p* < 0.01; *** *p* < 0.001; **** *p* < 0.0001.

***2. Pearson correlation analysis to explore the potential associations between intestinal flora and fecal biomarkers***


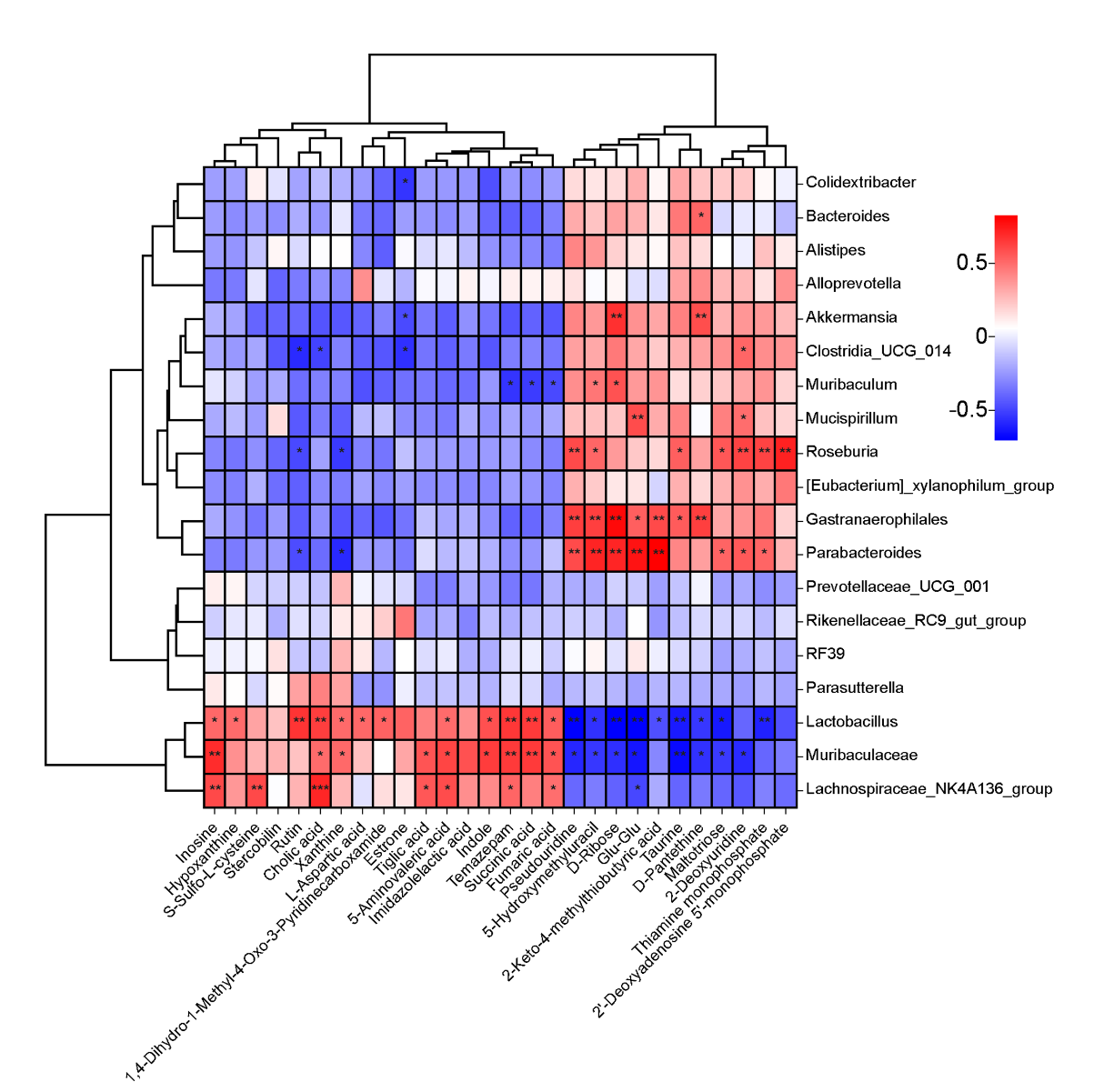


**Figure S2**. Correlation analysis between intestinal microbiota and fecal biomarkers heat map. Red represents positive correlation, and blue indicates negative correlation. **p* < 0.05; ** *p* < 0.01; *** *p* < 0.001.
